# Average Values and Racial Differences of Neutrophil Lymphocyte Ratio among a Nationally Representative Sample of United States Subjects

**DOI:** 10.1371/journal.pone.0112361

**Published:** 2014-11-06

**Authors:** Basem Azab, Marlene Camacho-Rivera, Emanuela Taioli

**Affiliations:** 1 Department of Surgery, Staten Island University Hospital, Staten Island, New York, United States of America; 2 Department of Population Health, North Shore LIJ-Hofstra School of Medicine, Great Neck, New York, United States of America; The Ohio State University, United States of America

## Abstract

**Introduction:**

Several studies reported the negative impact of elevated neutrophil/lymphocyte ratio (NLR) on outcomes in many surgical and medical conditions. Previous studies used arbitrary NLR cut-off points according to the average of the populations under study. There is no data on the average NLR in the general population. The aim of this study is to explore the average values of NLR and according to race in adult non-institutional United States individuals by using national data.

**Methods:**

The National Health and Nutrition Examination Survey (NHANES) of aggregated cross-sectional data collected from 2007 to 2010 was analyzed; data extracted included markers of systemic inflammation (neutrophil count, lymphocyte count, and NLR), demographic variables and other comorbidities. Subjects who were prescribed steroids, chemotherapy, immunomodulators and antibiotics were excluded. Adjusted linear regression models were used to examine the association between demographic and clinical characteristics and neutrophil counts, lymphocyte counts, and NLR.

**Results:**

Overall 9427 subjects are included in this study. The average value of neutrophils is 4.3k cells/mL, of lymphocytes 2.1k cells/mL; the average NLR is 2.15. Non-Hispanic Black and Hispanic participants have significantly lower mean NLR values (1.76, 95% CI 1.71–1.81 and 2.08, 95% CI 2.04–2.12 respectively) when compared to non-Hispanic Whites (2.24, 95% CI 2.19–2.28–p<0.0001). Subjects who reported diabetes, cardiovascular disease, and smoking had significantly higher NLR than subjects who did not. Racial differences regarding the association of smoking and BMI with NLR were observed.

**Conclusions:**

This study is providing preliminary data on racial disparities in a marker of inflammation, NLR, that has been associated with several chronic diseases outcome, suggesting that different cut-off points should be set according to race. It also suggests that racial differences exist in the inflammatory response to environmental and behavioral risk factors.

## Introduction

Inflammation plays a major role in the pathophysiology of commonly considered non-inflammatory diseases, such as cancer and atherosclerosis [1]–[4]. Among many inflammatory markers, several studies demonstrated that elevated neutrophil/lymphocyte ratio (NLR) is a significant predictor of adverse outcomes for patients with cardiovascular disease or cancer [5]–[8]. NLR is believed to reflect the balance between innate (neutrophils) and adaptive (lymphocytes) immune responses. Previous research has shown that elevated NLR is associated with increased concentration of various pro-inflammatory cytokines [8]–[10] which may cause cellular DNA damage.

These studies corroborate the negative impact of elevated NLR, however they differ in their NLR cutoff points. While some studies categorized their patients according to NLR intervals (e.g. tertiles, quartiles, quintiles) [11]–[13], other studies used definite NLR cutoff points (e.g. NLR≥2.5 [14], NLR≥2.7 [15], NLR≥3 [16], NLR≥4 [17], and others used NLR≥5 [18]–[20]. Of note, the studies from western countries often used higher NLR cutoff points compared to other ethnicity (e.g. Asian and African), which reflect well known racial difference in the normal range of neutrophil and lymphocyte counts [21], [22]. It is not known, however, if differences observed in NLR reflect real variation among healthy human subjects, or are related to the lack of standardization in the measurement of this biomarker. In fact, studies report differ timing for the collection of blood used to calculate NLR; some collect the blood sample on admission [23], others use preoperative NLR [24], maximum NLR during hospitalization [13], or average NLR of three readings during hospitalization [25]. Nevertheless, there is no study to our knowledge exploring the normal range and variability of NLR in a healthy population. Aim of this study was to investigate the normal range of NLR and its relationship with other demographic, risk factor and comorbidity variables in a well-known maintained national database of non-institutional individuals (NHANES).

## Methods

### Study design and participants

The National Health and Nutrition Examination Survey (NHANES) is a population-based survey designed to assess the health and nutritional status of non-institutionalized children and adults in the United States. NHANES uses a complex, multistage, probability sampling design to produce a nationally representative sample of non-institutionalized US children and adults. In this study, we aggregated cross-sectional data collected from 2007 to 2010; data extracted included markers of systemic inflammation (neutrophil count, lymphocyte count, and NLR), demographic (age, sex, race, Body Mass Index) and clinical (history of diabetes, heart disease or heart attack) characteristics.

In the 2007–2008 NHANES survey, there were 8249 subjects of both sexes, aged ≥18 years, who had complete data on neutrophil or lymphocyte counts; 3,427 participants were excluded for reporting a history of cancer or malignancy or missing data on cancer or malignancy. An additional 279 participants were excluded for self- report of taking any of the following medications: steroids, chemotherapy, immunomodulators, antibiotics, leaving 4,548 subjects (55% of the original sample). In the 2009–2010 survey, the same exclusion criteria were applied; from the initial 8,786 men and women who had neutrophil and lymphocyte data, a total of 4,884 participants (approximately 56% of the original sample) were included in the present analysis. The final sample consisted of 9,427 subjects across both survey waves.

### NHANES Data collection and laboratory analysis

Data collected regarding demographic information (age, race, education, health insurance status, and income to poverty ratio), current medication use, diagnosis of medical conditions (both previous and current), and lifestyle behaviors (smoking and alcohol use) were collected by trained interviewers. Body Mass Index (BMI) (kg/m^2^) was measured during the medical examination. Laboratory tests were performed on collected blood specimens to provide information on neutrophil count (1,000 cells/µl) and lymphocyte count (1,000 cells/µl). NLR was calculated as the ratio of neutrophil cell count to lymphocyte cell count. The Coulter method was used to determine neutrophil and lymphocyte counts (Coulter Gen.S Hematology Analyzer, Beckman Coulter Corp, Hialeah, Florida).

### Data analysis

The distribution of continuous variables was reported as means ± standard deviation, of categorical variables as frequencies and percentages. The main outcome of interest, neutrophil, lymphocyte counts and the NLR was reported as mean along with 95% confidence intervals. To examine the influence of demographic and clinical characteristics on neutrophil counts, lymphocyte counts, and NLR, linear regression models for each outcome were performed, including all the variables found to be statistically significant at univariate analysis (p<0.05) as well as clinically meaningful. Multivariate linear regression models were also stratified by race. All data analyses used the appropriate survey sample weights to provide nationally representative estimates. Statistical significance was determined at alpha level of 0.05. Analyses were performed using Stata/SE version 12 (StataCorp. 2011. *Stata Statistical Software: Release 12*. College Station, TX: StataCorp LP).

## Results

There are 9427 subjects in this study (Table 1), distributed equally between males and female. The majority of subjects is white (67%), has completed at least high school or more (55%), and is covered by some form of insurance (78%). The average age is 47 years; roughly one third of the subjects is overweight (BMI≥30 km/m^2^). The rate of comorbidities varies from 4% (heart condition) to 7% (diabetes). Forty four percent of subjects are classified as ever smokers, while 76% indentified themselves ad current drinkers. The average value of neutrophils is 4.27×1000 cells/µL, of lymphocytes 2.14×1000 cells/µL; the average NLR is 2.15.

**Table 1:** Sample characteristics among NHANES 2007–2010 participants (n = 9427).

Variable	Categories	Subjectstested (N)	WeightedPrevalence(95% CI)
**Race**	Hispanic	2904	14.72 (10.92–18.53)
	Non-Hispanic White	4270	67.11 (61.77–72.44)
	Non-Hispanic Black	1756	11.03 8.92–13.14)
	Other Non-Hispanic	497	07.14 (5.46–8.82)
**Sex**	Male	4625	49.03 (47.95–50.11)
	Female	4802	50.97 (49.89–52.04)
**Age (years)**		9427	47.56 (46.92–48.21)
**Education**	< high school	2842	19.96 (18.15–21.77)
	High school or equiv	2246	24.09 (22.31–25.88)
	> high school	4325	55.84 (53.12–58.57)
**Income/poverty ratio**		8548	2.97 (2.88–3.07)
**Health insurance**	Insured	6871	78.33 (76.56–80.10)
**Diabetes**	Yes	1044	7.64 (6.69–8.60)
	Borderline	162	1.6 (1.25–1.86)
**Heart condition**	Yes	517	4.06 (3.57–4.55)
**BMI (kg/m^2^)**	<18.5	140	1.64 (1.20–2.10)
	18.5–24.9	2469	29.20 (27.60–30.79)
	25.0–29.9	3167	33.82 (32.37–35.27)
	≥30.0	3562	35.34 (33.78–36.90)
**Ever Smoker**	Yes	4322	44.85 (42.37–47.34)
**Current Drinker**	Yes	6213	76.64 (74.43–78.55)
			**Mean (95% CI)**
**Segmented** **Neutrophils** **(1000 cells/µL)**		9427	4.27 (4.20–4.34)
**Lymphocytes** **(1000 cells/µL)**		9427	2.14 (2.11–2.16)
**Neutrophil/lymphocyte** **ratio (NLR)**		9427	2.15 (2.11–2.19)

NLR was studied in relation to personal and demographic variables (Table 2). Non-Hispanic Black participants and Hispanic participants had significantly lower mean NLR values (1.76, 95% CI 1.71–1.81 and 2.08, 95% CI 2.04–2.12 respectively) when compared to non-Hispanic Whites (mean NLR = 2.24, 95% CI 2.19–2.28–p<0.0001). Similar results were observed in children (table S1). Subjects who reported diabetes or a history of heart condition had higher NLR than subjects who did not, but only results on hearth condition were statistically significant (p<0.0001). Ever smokers had significantly higher NLR than non smokers (p = 0.001). There were no significant differences in NLR with sex, education, insurance status, or drinking habits. There was a significant trend of increasing NLR with increasing BMI (p for trend: 0.002). The associations persisted after adjustment for confounding factors (table 3). In addition NLR was significantly associated with increasing age and inversely associated with income poverty ratio.

**Table 2:** Neutrophil, Lymphocyte, and NLR according to demographic and clinical characteristics (n = 9427).

Variable	Categories	NeutrophilMean (95% CI)	LymphocyteMean (95% CI)	NLRMean (95% CI)
**Race**	Hispanic	4.39 (4.31–4.48)	2.26 (2.23–2.29)	2.08 (2.04–2.12)
	Non-Hispanic White	4.35 (4.27–4.44)	2.09 (2.06–2.12)	2.24 (2.19–2.28)
	Non-Hispanic Black	3.65 (3.56–3.73)	2.24 (2.21–2.28)	1.76 (1.71–1.81)
	Other Non-Hispanic	4.18 (4.00–4.37)	2.11 (2.05–2.17)	2.10 (2.01–2.19)
**Sex**	Male	4.26 (4.18–4.33)	2.11 (2.08–2.13)	2.19 (2.14–2.25)
	Female	4.28 (4.20–4.36)	2.16 (2.14–2.19)	2.11 (2.07–2.16)
**Education**	< high school	4.44 (4.32–4.56)	2.22 (2.19–2.26)	2.16 (2.09–2.24)
	High school or equiv	4.45 (4.34–4.56)	2.19 (2.15–2.23)	2.20 (2.14–2.26)
	> high school	4.13 (4.07–4.20)	2.08 (2.06–2.10)	2.13 (2.08–2.17)
**Health insurance**	Insured	4.21 (4.14–4.28)	2.10 (2.08–2.12)	2.16 (2.11–2.21)
	Uninsured	4.48 (4.37–4.60)	2.26 (2.21–2.30)	2.12 (2.06–2.18)
**Diabetes**	Yes	4.66 (4.50–4.83)	2.21 (2.15–2.27)	2.34 (2.23–2.45)
	No	4.24 (4.17–4.30)	2.13 (2.11–2.15)	2.13 (2.09–2.17)
	Borderline	4.27 (3.88–4.67)	2.06 (1.97–2.15)	2.21 (2.01–2.41)
**Heart condition**	Yes	4.26 (4.19–4.33)	2.14 (2.12–2.16)	2.44 (2.30–2.58)
	No	4.45 (4.28–4.61)	2.03 (1.93–2.12)	2.14 (2.10–2.18)
**BMI (kg/m^2^)**	<18.5	3.99 (3.64–4.34)	2.10 (1.97–2.23)	2.06 (1.85–2.27)
	18.5–24.9	3.97 (3.88–4.07)	2.01 (1.98–2.05)	2.11 (2.06–2.17)
	25.0–29.9	4.18 (4.08–4.28)	2.11 (2.08–2.15)	2.13 (2.07–2.20)
	≥30.0	4.62 (4.54–4.71)#	2.26 (2.23–2.29)#	2.21 (2.15–2.27)*
**Ever Smoker**	Yes	4.55 (4.43–4.66)	2.21 (2.19–2.24)	2.22 (2.16–2.28)
	No	4.04 (4.00–4.09)	2.07 (2.04–2.10)	2.10 (2.05–2.14)
**Drinker**	Yes	4.28 (4.21–4.35)	2.13 (2.10–2.16)	2.16 (2.11–2.20)
	No	4.22 (4.11–4.33)	2.12 (2.08–2.16)	2.17 (2.09–2.25)

*P for trend: 0.002;

# p for trend<0.0001.

**Table 3:** Adjusted Linear Regression Estimates of the association between demographic and clinical characteristics and Neutrophil and Lymphocyte Values (n = 7736).

		Neutrophil			Lymphocyte			NLR		
Variable	Categories	Coeff	95% CI	p-value	Coeff	95% CI	p-value	Coeff	95% CI	p-value
**Race**	Hispanic	−0.14	(−0.27−−0.01)	0.03	0.09	(0.05–0.13)	<0.0001	−0.18	(−0.26−−0.11)	<0.0001
	Non-Hispanic White	REF			REF			REF		
	Non-Hispanic Black	−0.92	(−1.03 −−0.81)	<0.0001	0.10	(0.06–0.15)	<0.0001	−0.55	(−0.61−−0.49)	<0.0001
	Other Non-Hispanic	−0.02	(−0.21−0.24)	0.89	0.05	(−0.04–0.13)	0.24	−0.07	(−0.19−0.05)	0.24
**Sex**	Female vs Male	0.16	(0.06–0.26)	0.002	0.10	(0.07–0.14)	<0.0001	−0.06	(−0.13–0.006)	0.07
**Age (years)**	Continuous	−0.007	(−0.009–−0.004)	<0.0001	−0.006	(−0.007–−0.005)	<0.0001	0.004	(0.002–0.006)	<0.0001
**Education**	<high school	REF			REF			REF		
	High school or equiv	0.10	(−0.02–0.21)	0.09	−0.013	(−0.06–0.31)	0.55	0.05	(−0.04–0.14)	0.29
	>high school	−0.12	(−0.25–0.004)	0.06	−0.06	(−0.10–−0.03)	0.002	−0.01	(−0.09–0.06)	0.72
**Income/poverty ratio**	Continuous	−0.05	(−0.08–−0.02)	0.003	−0.01	(−0.02–0.003)	0.14	−0.03	(−0.05–0.007)	0.01
**Health insurance**	Uninsured vs Insured	0.20	(0.02–0.38)	0.03	0.05	(−0.07–0.10)	0.09	0.02	(−0.06–0.10)	0.61
**Diabetes**	Yes	0.32	(0.15–0.48)	<0.0001	0.08	(0.02–0.14)	0.009	0.11	(−0.004–0.23)	0.06
	No	REF			REF			REF		
	Borderline	−0.16	(−0.56–0.23)	0.40	−0.12	(−0.24–−0.11)	0.03	−0.006	(−0.22–0.21)	0.95
**Heart condition**	Yes vs No	.06	(−.13–.24)	0.55	−0.09	(−0.21–0.03)	0.13	0.17	(0.05–0.30)	0.008
**BMI (kg/m^2^)**	<18.5	0.04	(−0.37–0.45)	0.85	0.04	(−0.10–0.17)	0.59	0.008	(−0.22–0.24)	0.94
	18.5–24.9	REF			REF			REF		
	25.0–29.9	0.24	(0.09–0.38)	0.003	0.12	(0.07–0.16)	<0.0001	−0.005	(−0.10–0.09)	0.91
	≥30.0	0.71	(0.57–0.85)	<0.0001	0.27	(0.23–0.31)	<0.0001	0.08	(0.001–0.15)	0.046
**Ever Smoker**	Yes vs No	0.46	(0.33–0.60)	<0.0001	0.15	(0.12–0.19)	<0.0001	0.09	(0.02–0.16)	0.015
**Drinker**	Yes vs No	−0.02	(−0.14–0.10)	0.73	0.005	(−0.04–0.05)	0.81	−0.03	(−0.13–0.07)	0.55

When the analysis was repeated according to race (Table 4), among black subjects a high NLR was significantly associated only with increasing BMI (β coefficient = 0.15, 95% CI 0.0005–0.29). Among non-Hispanic Whites, older age (p<0.0001) and being a smoker (p = 0.04) were positively associated with increasing NLR values, while income to poverty ratio was negatively associated with NLR (p = 0.01); women had significantly lower NLR values compared to men (β coefficient =  −0.10, 95% CI −0.19– −0.02; p = 0.01). Among Hispanics, only having a heart condition was significantly associated with an increased NLR (β coefficient = 0.21, 95% CI 0.002–0.42; p = 0.04).

**Table 4:** Linear Regression Estimates (β coefficients and 95% CI) of the association between clinical and demographic characteristics and NLR according to racial subgroups.

Variable	Categories	Blacks(n = 1440)	Whites(n = 3684)	Hispanics(n = 2255)
**Sex**	Female vs Male	−0.09 (−0.19–0.007)	−0.10 (−0.19– −0.02)∧	0.04 (−0.05–0.13)
**Age (years)**	Continuous	0.001 (−0.001–0.005)	0.005 (0.002–0.007) #	0.002 (−0.0001–0.006)
**Education**	<high school	REF	REF	REF
	High school	0.08 (−0.05–0.21)	− 0.001 (−0.13–0.13)	0.10 (−0.03–0.23)
	>high school	0.06 (−.08–0.19)	−0.06 (−0.18–0.05)	0.02 (−0.04–0.09)
**Income/poverty ratio**	Continuous	−0.02 (−0.05–0.02)	−0.03 (−0.06– −0.008)^ ∧^	−0.03 (−0.06–0.003)
**Health insurance**	Uninsured vs Insured	−0.05 (−0.16–0.05)	0.02 (−0.10–0.13)	−0.02 (−0.13–0.08)
**Diabetes**	Yes	0.16 (−0.04–0.37)	0.15 (−0.05–0.34)	−0.02 (−0.13–0.09)
	No	REF	REF	REF
	Borderline	0.33 (−0.01–0.66)	−0.05 (−0.33–0.24)	−0.10 (−0.28–0.09)
**Heart condition**	Yes vs no	0.11 (−0.11–0.33)	0.15 (−0.01–0.31)	0.21 (0.002–0.42)*
**BMI (kg/m^2^)**	<18.5	0.12 (−0.50–0.73)	−0.02 (−0.31–0.28)	−0.11 (−0.92–0.69)
	18.5–24.9	REF	REF	REF
	25.0–29.9	−0.001 (−0.19–0.19)	−0.01 (−0.13–0.11)	−0.01 (−.12–0.11)
	≥30.0	0.15 (0.0005–0.29)*	0.05 (−0.06–0.15)	0.07 (−.05–0.20)
**Ever Smoker**	Yes vs no	0.02 (−0.08–0.12)	0.09 (0.002–0.18)*	0.01 (−0.07–0.10)
**Drinker**	Yes vs no	0.06 (−0.03–0.15)	−0.06 (−0.20–0.08)	0.06 (−0.04–0.16)

*p = 0.04;

# p<0.0001;

∧p = 0.01.

## Discussion

The present analysis of a large US data set including over 9000 subjects reports the average value for NLR in the general population, and indicates that such normal value significantly varies with race; NLR is particularly low in Non-Hispanic Black subjects, from 2.24 observed in Whites to 1.76 in Blacks. This finding has important clinical implications. Several publications demonstrated that an elevated NLR is a predictor of poor outcome in cancer [7] and cardiovascular disease [5]; these studies however used arbitrary NLR cut off points for risk stratification, which were based on the average NLR values of each study population. Such populations were mostly small in size, without consideration of racial differences and racial composition.

Because of the lower NLR observed in black in comparison to white subjects, it is possible that commonly reported high prognostic NLR cut-off points be hardly reached by non-white populations, or be a much worst prognostic indicator than in white patients. All these speculations need to be tested in multi ethnic populations affected by chronic diseases such as cancer and cardiovascular disease.

Another result of this analysis is that NLR is associated with several self-reported chronic conditions, such as diabetes and heart disease, with being a smoker, with high BMI, and with increasing age, all conditions that are known to increase the body inflammatory milieu [26]. In addition this study shows that an index of socioeconomic status, the income to poverty ratio, is inversely associated with NLR. A low socio economic status may be a proxy for poor dietary habits, low in nutrients and anti-oxidants, or lack of physical exercise, or occupational exposures to chemicals and carcinogens. We are not able to test these hypotheses given the retrospective nature of the NHANES data base, and the limited information available from the questionnaire.

This analysis also shows that the association between personal and behavioral factors and NLR differs with race. For example, among black patients only BMI was significantly associated with elevated NLR, while among white patients several expected factors, such as age and smoking habits were associated with higher NLR. These differences may be due to chance, or to a different host response to pro-inflammatory factors, a hypothesis that needs to be tested in *ad hoc* studies.

Despite the fact that this analysis relies on a large sample of the US population, a larger sample size collected over longer periods of time would help better defining NLR normal ranges. In addition, the NLR was an occasional, single measure, and as such does not reflect individual variability. Another limitation is that all the exposure variables, including race were self-reported, thus their accuracy could not be objectively verified. However, it is unlikely that the answers to the questionnaire could differ according to the NLR, since the participants were not aware of the results of the test.

This study is providing preliminary data on racial disparities in a marker of inflammation (NLR) that has been associated with several chronic diseases outcome, suggesting that different cut-off points should be set according to race. It also shows how NLR is associated with personal and behavioral factors, some of which are modifiable such as smoking and BMI. It suggests that together with public health interventions of the factors amenable of being modified, chemopreventive trials should be considered in an attempt to modify NLR in ageing people, smokers and populations at risk for chronic diseases such as cardiovascular disease and cancer.

## Conclusions

The study indicates racial differences of average NLR among non-Hispanic black, Hispanic and non-Hispanic white subjects, with average NLR of 1.76, 2.08 and 2.24, respectively. The results corroborate prior studies on inflammation in reporting the association between elevated NLR and risk factors such as smoking, obesity, and diabetes. Moreover, differences in the association between some of these risk factors and elevated NLR across different races were observed. This may illustrate racial differences in inflammatory response to different risk factors, some of which are modifiable.

## Supporting Information

Table S1 **Mean Neutrophil, Lymphocyte, and NLR values according to demographic and clinical characteristics for children age 2–18 years (n = 5286) - NHANES data set.**
(DOCX)
