# Supplementary material for: Average Values and Racial Differences of Neutrophil Lymphocyte Ratio among a Nationally Representative Sample of United States Subjects
Source: PLoS One. 2014 Nov 6;9(11):e112361. doi: 10.1371/journal.pone.0112361 (PMC4223021; doi:10.1371/journal.pone.0112361)
Supplement: Table S1 — Mean Neutrophil, Lymphocyte, and NLR values according to demographic and clinical characteristics for children age 2–18 years (n = 5286) - NHANES data set. (DOCX) [file pone.0112361.s001.docx]

| **Table S1. Mean Neutrophil, Lymphocyte, and NLR values according to demographic and clinical characteristics for children age 2-18 years (n = 5286)- NHANES data set** | | | | |
| --- | --- | --- | --- | --- |
| **Variable** | **Categories** | **Neutrophil**  **Mean (95% CI)** | **Lymphocyte**  **Mean (95% CI)** | **NLR**  **Mean (95% CI)** |
| **Race/ethnicity** | Hispanic | 4.02 (3.92-4.14) | 2.75 (2.71-2.79) | 1.63 (1.57-1.69) |
|  | Non-Hispanic White | 3.66 (3.53-3.79) | 2.55 (2.51-2.60) | 1.58 (1.52-1.65) |
|  | Non-Hispanic Black | 3.02 (2.93-3.11) | 2.63 (2.57-2.69) | 1.26 (1.22-1.31) |
|  | Other Non-Hispanic | 3.42 (3.23-3.62) | 2.79 (2.67-2.91) | 1.37 (1.26-1.47) |
| **Sex** | Male | 3.50 (3.40-3.60) | 2.58 (2.54-2.61) | 1.49 (1.44-1.55) |
|  | Female | 3.78 (3.67-3.89) | 2.68 (2.62-2.73) | 1.58 (1.53-1.62) |
| **BMI (Kg/m^2^)** | Normal | 3.42 (3.31-5.53) | 2.66 (2.61-2.70) | 1.44 (1.38-1.49) |
|  | Overweight | 3.80 (3.68-3.92) | 2.48 (2.42-2.54) | 1.67 (1.59-1.76) |
|  | Obese | 4.33 (4.13-4.52) | 2.67 (2.60-2.75) | 1.74 (1.64-1.83) |
| **Asthma** | Yes | 3.72 (3.56-3.87) | 2.60 (2.52-2.67) | 1.58 (1.50-1.66) |
|  | No | 3.62 (3.54-3.71) | 2.63 (2.60-2.66) | 1.52 (1.59-1.57) |
